# Modeling Pharmacodynamic Response to the Poly(ADP-Ribose) Polymerase Inhibitor ABT-888 in Human Peripheral Blood Mononuclear Cells

**DOI:** 10.1371/journal.pone.0026152

**Published:** 2011-10-10

**Authors:** Jiuping Ji, Robert J. Kinders, Yiping Zhang, Larry Rubinstein, Shivaani Kummar, Ralph E. Parchment, Joseph E. Tomaszewski, James H. Doroshow

**Affiliations:** 1 National Clinical Target Validation Laboratory, National Cancer Institute at Frederick, Frederick, Maryland, United States of America; 2 Laboratory of Human Toxicology and Pharmacology, Applied/Developmental Research Support Directorate, SAIC-Frederick, Inc., National Cancer Institute at Frederick, Frederick, Maryland, United States of America; 3 Division of Cancer Treatment and Diagnosis, National Cancer Institute, Bethesda, Maryland, United States of America; 4 Center for Cancer Research, National Cancer Institute, Bethesda, Maryland, United States of America; Roswell Park Cancer Institute, United States of America

## Abstract

**Background:**

Poly(ADP-ribose) polymerase (PARP)facilitates DNA repair and PARP inhibitors may potentiate the effect of DNA-damaging chemotherapeutic agents in patients with cancer. Collection of peripheral blood mononuclear cells (PBMCs)as a surrogate tissue to monitor PARP inhibitor pharmacodynamic effects has several advantages over tumor biopsy collection, including minimally invasive sample collection and the ability to collect multiple samples for longitudinal assessment of drug effect.

**Methodology/Principal Findings:**

Using our previously validated immunoassay for measuring poly(ADP-ribose) (PAR), a product of PARP, in tumor biopsies, we validated a method to quantify PAR levels in PBMCs to monitor the pharmacodynamic effects of the PARP inhibitor ABT-888 in clinical trials. The inter-individual variation in PAR levels was large. No significant difference (*P* = 0.67) was measured between median baseline PAR levels in 144 healthy volunteers (131.7 pg/1×10^7^ PBMCs [interquartile range, 79.5–241.6]) and 49 patients with cancer (149.2 pg/1×10^7^ PBMCs [interquartile range, 83.2–249.3]). In addition, PAR levels monitored in healthy volunteers over 3 weeks had considerable intra- and inter-individual variation (range, 44–1073 pg PAR/1×10^7^ PBMCs). As a pharmacodynamic model, we quantified changes in PAR levels in human PBMCs treated ex vivo with clinically relevant concentrations of ABT-888. Of 40 healthy volunteer PBMC samples treated with ABT-888, 47.5% had greater than 50% PAR reduction compared to vehicle-treated controls. Considerable inter-sample heterogeneity in PAR levels was measured, and several ABT-888–insensitive samples were identified.

**Conclusions/Significance:**

Our results emphasize the importance of using a validated method to measure PAR levels, and support further investigation into the role of PARP in PBMCs. To this end, the PAR immunoassay has been validated for use with PBMCs and incorporated into clinical trials to assess PBMCs as a potential pharmacodynamic surrogate for tumor biopsies in clinical trials of PARP inhibitors.

## Introduction

The National Cancer Institute (NCI) initiated a Phase 0 clinical trial and pharmacodynamic assay program to demonstrate target inhibition of poly(ADP-ribose) polymerase 1 (PARP1) by ABT-888, a potent, orally available PARP inhibitor, in tumor biopsies and peripheral blood mononuclear cells (PBMCs) from patients with advanced malignancies [1]. Because PARP enzymes are essential for DNA damage recognition and base excision repair, PARP inhibitors such as ABT-888 have considerable potential as chemotherapeutic agents [2]–[7]. Critical to the conduct of the Phase 0 trial was validation of an immunoassay for poly(ADP-ribose) (PAR), the product of PARP1, that was sufficiently sensitive, reproducible, and accurate to measure drug-induced modulation of PAR levels in tumor and PBMC samples under clinically relevant conditions [8]–[11]. Human, mouse, and rat preclinical tumor models were previously employed to validate a method to measure PAR levels and model the pharmacodynamics of ABT-888 in human tumor tissues [3], [8], [12]; however, there is no equivalent mouse model for whole blood. The advantages of using whole blood include straightforward and minimally invasive sample collection, a relatively large sample volume, and the ability to collect multiple specimens over time. To determine whether ABT-888 would exert a comparable effect on PAR levels in PBMCs as in tumors, we adapted the PAR immunoassay used for tumor tissue [8], [10] and validated the method for PBMCs using an ex vivo human PBMC model and standard clinical chemistry methods. This assay was subsequently incorporated in early-phase clinical trials of ABT-888 and other PARP inhibitors. Our interest was to use the PAR immunoassay to explore the potential of PBMCs as pharmacodynamic surrogates for PAR response in tumor biopsy samples.

## Results

### Method development and validation

**Figure 1:**
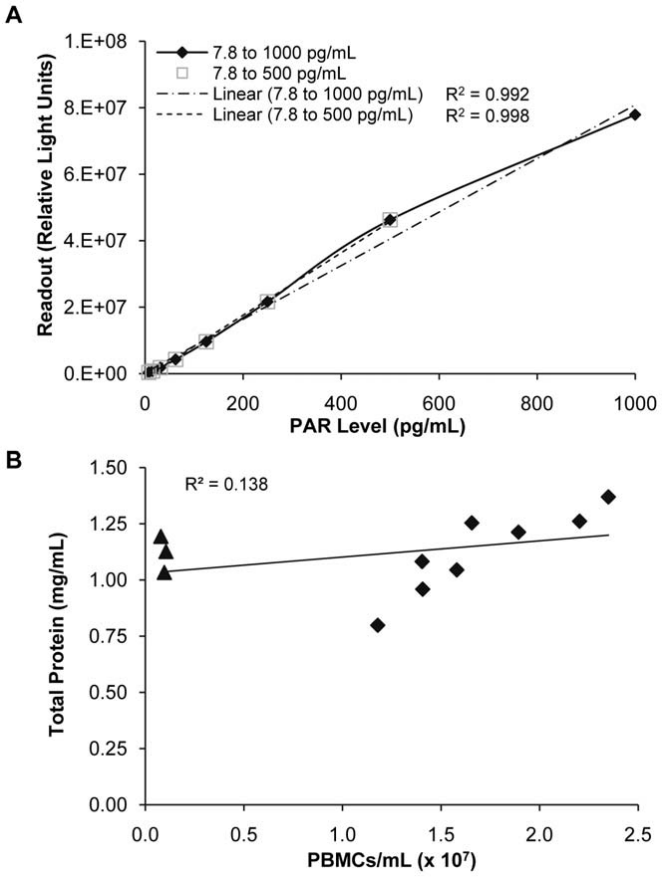
Dilution linearity of the PAR polymer standards and protein content from isolated PBMC samples. (A) Dilution linearity of the PAR polymer standards was determined during quantitative validation of the PAR immunoassay for PBMCs. Concentrations of PAR standards ranged from 7.8 to 1000 pg PAR/mL. (B) Total protein content (bicinchoninic acid protein assay) for isolated PBMCs (counted by hemocytometer) from 11 healthy volunteers. Triangles indicate samples where plasma protein contamination skewed the total protein readout when compared to cell number.

Our laboratory has modified and cross-validated a PAR immunoassay for tumor biopsies to quantify PAR levels in isolated human PBMC samples. Critical reagents validated for the PAR immunoassay for tumor biopsies were tested and used in the assay reported herein, including the rabbit polyclonal PAR antibody, rabbit monoclonal PAR antibody, and assay standards [8], [10]. Dilution linearity of the PAR polymer standards was assessed and resulted in an adjusted R^2^ value of 0.992 over the 7.8 to 1000 pg PAR/mL range (Figure 1A); the slope of the curve of PAR readout in the immunoassay decreased by 75% above 1000 pg PAR/mL (data not shown). The PAR immunoassay dynamic range for PBMCs was set at 7.8 to 1000 pg PAR/mL, with the lower limit of quantitation and lower limit of detection determined within each assay run.

Although PAR levels were measurable in mouse PBMCs and splenocytes in preliminary studies with a B16–F10 murine melanoma xenograft model, treatment with ABT-888 reduced PAR levels below the assay lower limit of detection (data not shown). In addition, collecting sufficient volumes of mouse PBMCs for longitudinal assessment of PARP inhibition was impractical; therefore, an ex vivo human PBMC model was developed. Unlike the validated PAR immunoassay for tumor biopsies, where sample input is normalized to protein concentration [8], [10], samples for the PBMC immunoassay were normalized to PBMC number. When total protein content for samples with increasing PBMCs/mL was measured, contamination by plasma proteins resulted in PBMC samples with as few as 0.08×10^7^ cells/mL having a total protein content readout equal to that seen in samples with 1.89×10^7^ cells/mL (1.20 mg/mL and 1.21 mg/mL protein, respectively; Figure 1B). Samples prepared for the PAR immunoassay based on these protein concentrations would give low final PAR readouts due to lack of cellular protein rather than inherently low PAR levels. Analysis of increasing PBMC concentrations with the PAR immunoassay demonstrated a positive correlation in PAR recovery in the range of 2×10^6^ to 5×10^7^ cells/mL; higher cell concentrations resulted in viscosity issues due to DNA contamination (data not shown). Therefore, a concentration of 1×10^7^ viable PBMCs/mL was used to standardize the sample input for the assay.

Quantitative validation of the chemiluminescent immunoassay for PAR in PBMCs was carried out to establish assay accuracy and precision. Assay accuracy was determined by comparison of expected to actual recovered levels of PAR in healthy volunteer PBMC extracts spiked with PAR polymer. PAR recovery was calculated for three paired replicates assayed by two different assay operators; samples were run as unknowns and yielded a total assay accuracy of 103.3%±11.7% (mean ± SD; Table 1). Assay precision testing measured inter-operator and inter-day variability using PBMC extracts spiked with PAR polymer (31.25, 62.5, and 125 pg PAR/mL) and control samples (Colo829 human melanoma extracts). All samples were run as unknowns by two operators, on two different luminometers, on 3 different days and read against a PAR polymer standard curve to determine PAR concentration. The intra-assay coefficient of variation (CV) for the two operators ranged from 3.6% to 19.4%, and inter-plate CVs ranged from 5.2% to 19.5% (Table 2). Additional precision data were collected from seven PAR immunoassay training courses [10] held by the Division of Cancer Treatment and Diagnosis at NCI-Frederick (October 2008–April 2011); these courses included a total of 19 student trainees and 18 healthy volunteer PBMC samples. For each training course, two to three PBMC samples were analyzed by two to four student trainees; in four of the courses, the trainer ran a plate in parallel with the students. Relative PAR levels were determined for each sample and compared across days and operators to determine overall assay precision. The mean intra-plate CV for all student trainee runs was 6.1% (data not shown), and the mean intra-operator CV was 6.7% over the seven courses (range, 1.2%–26.2%; Table 3). Using readouts from the control samples of the 19 student trainees during their 3-day course, student assay imprecision was 22.6% (data not shown). Data from four plates run by the trainer were also examined; the trainer had a mean intra-operator CV of 14.7% and an assay imprecision of 18.5% (data not shown).

**Table 1:** Recovery of PAR from spiked PBMC extracts.

	PAR immunoassay readout (pg/mL)				
Operator-replicate	Intrinsic PAR	Spiked PAR polymer	Expected recovery	Actual recovery	Recovery (%)
OP1-1	112	31	143	134	93.7
OP2-1	112	63	175	154	88.0
OP1-2	144	125	269	260	96.7
OP2-2	98	250	348	394	113.2
OP1-3	229	125	354	406	114.7
OP2-3	229	250	479	538	112.3
				**Mean percent recovery ± SD**	103.3±11.7

Abbreviations: OP, operator; SD, standard deviation.

**Table 2:** Intra- and inter-plate precision determined with PAR-spiked PBMC extracts and control cell lines.

	Operator 1: Intra-plate CV (%)			Operator 2: Intra-plate CV (%)			
	Day 1^a^	Day 2^a^	Day 3^a^	Day 1^a^	Day 2^a^	Day 3^a^	Mean inter-plate CV (%)
Extract +31.25 pg PAR/mL^b^	7.1	6	9.8	6.7	15.4	19.4	14.5
Extract +62.5 pg PAR/mL^b^	7	9.9	4.7	4.8	14.2	5.9	9.7
Extract +125 pg PAR/mL^b^	4.8	4.6	7.4	7.5	16	13.7	10.5
Assay zero	14.4	12.3	5.4	7.7	5.9	5.6	13.7
Colo829-Low-2	6.9	6.9	3.6	–	–	–	18.6
Colo829-Low-1	–	–	–	11.1	18.8	11.8	19.5
Colo829-High-2	9.4	11.2	5.4	–	–	–	9.2
Colo829-High-1	–	–	–	8.4	13.9	7.3	5.2

Abbreviation: CV, coefficient of variation.

a Assays performed on 3 non-consecutive days.

b PBMC extracts were pooled and dilutions were spiked with known amounts of PAR polymer (31.25, 62.5, and 125 pg PAR/mL). Intra-plate CV was determined for triplicate repeats of each sample on each plate; inter-plate CV was calculated from all six plates. Samples were run as unknowns by the assay operators.

**Table 3:** Longitudinal comparison of PAR immunoassay training course PAR readout levels in PBMCs from healthy volunteers.

		Relative PAR (%)^a^					
Training date	Trainee^b^	PBMC sample 1	PBMC sample 2	PBMC sample 3	Mean	SD	Intra-operator CV (%)
October 27–29, 2008	OP1	93.2	92.3	90.3	91.9	1.5	1.6
	OP2	117.2	116.0	125.6	119.6	5.3	4.4
	OP3	89.6	91.7	84.1	88.5	4.0	4.5
December 10–12, 2008	OP1	102.6	112.1	113.1	109.3	5.8	5.3
	OP4	94.5	94.4	115.2	101.4	12.0	11.8
	OP5	102.9	93.5	71.7	89.3	16.0	17.9
August 17–19, 2009	OP6	128.5	121.8	ND	125.2	4.7	3.8
	OP7	75.8	75.9	87.1	79.6	6.5	8.2
	OP8	110.9	116.6	115.8	114.4	3.0	2.7
	OP9	84.8	85.7	97.1	89.2	6.9	7.7
October 28–30, 2009	OP10	101.8	99.1	106.6	102.5	3.8	3.7
	OP11	121.0	132.0	124.9	125.9	5.6	4.4
	OP12	83.5	81.9	88.6	84.7	3.5	4.2
	OP13	93.8	87.0	79.9	86.9	7.0	8.0
March 22–24, 2010	OP1	113.3	129.1	ND	121.2	11.2	9.2
	OP14	90.4	81.0	ND	85.7	6.6	7.7
	OP15	96.3	89.8	ND	93.1	4.6	4.9
July 19–21, 2011	OP16	101.6	43.3	ND	72.5	41.2	56.9^c^
	OP17	138.0	146.0	ND	142.0	5.7	4.0
	OP18	ND	240.0	ND	240	N/A	N/A
	OP19	60.4	87.9	ND	74.1	19.4	26.2
April 18–20, 2011	OP1	81.6	88.4	ND	85.0	4.8	5.7
	OP20	115.6	108.8	ND	112.2	4.8	4.3
	OP21	115.1	117.1	ND	116.1	1.4	1.2
	OP22	87.7	85.7	ND	86.7	1.4	1.6
					**Mean intra-operator CV (%)**		6.7

Abbreviations: CV, coefficient of variation; ND, not determined; OP, operator; SD, standard deviation; N/A, not applicable.

a Two to three different healthy volunteer PBMC samples were used for each training session.

b Trainee listed as OP1 in four of the seven sessions was the PAR immunoassay trainer.

c Outlier by Grubb's test (*P*<0.05); excluded from calculation of average intra-operator and inter-operator CVs.

### PAR levels in healthy volunteer and patient PBMC samples

**Figure 2:**
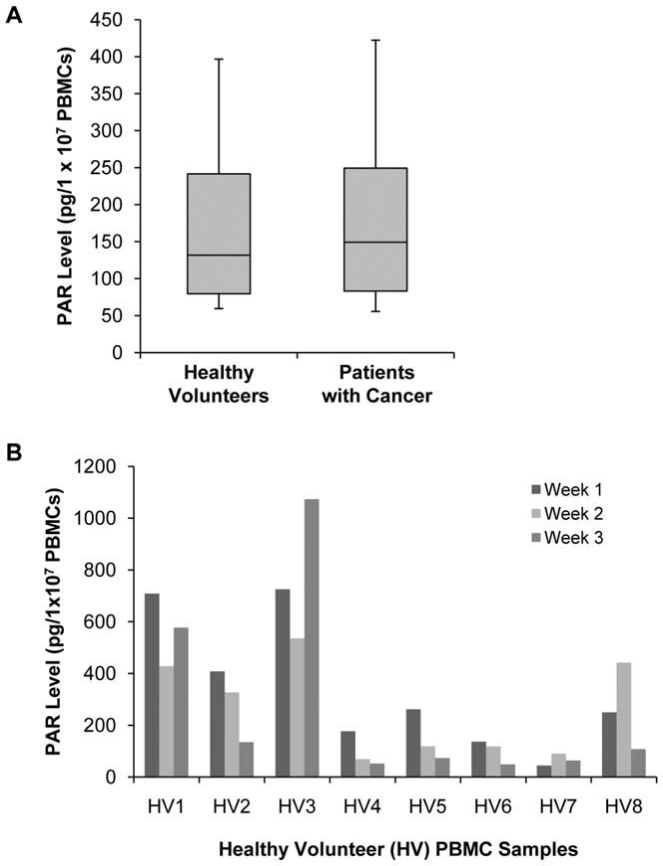
Baseline PAR levels in PBMCs from healthy volunteers and patients with cancer. (A) PAR levels in PBMC samples from 135 healthy volunteers and 47 patients with cancer. Box plot represents the interquartile range with median indicated; whiskers represent the 10^th^ and 90^th^ percentile. (B) PAR levels in PBMCs collected from eight healthy volunteers (HV) once per week for 3 consecutive weeks.

To determine whether baseline PAR levels in PBMCs differed between individuals with and without cancer, PAR levels were measured in samples from 144 healthy volunteers and 49 patients with cancer. PAR levels were above the lower limit of quantitation in 135 (94%) of the samples from healthy volunteers and 47 (96%) of the samples from patients with cancer. PAR levels ranged from 34 to 1322 pg PAR/1×10^7^ cells in PBMC samples from healthy volunteers and 31 to 1004 pg PAR/1×10^7^ cells in PBMC samples from patients with cancer (data not shown). The median PAR level in PBMC samples from healthy volunteers was 131.7 pg/1×10^7^ cells (interquartile range, 79.5–241.6) and in PBMC samples from patients with cancer was 149.2 pg/1×10^7^ cells (interquartile range, 83.2–249.3; Figure 2A). There was no statistical difference in PAR levels between the two groups (*P* = 0.67, Student's *t*-test).

PAR levels in PBMCs collected once per week for 3 consecutive weeks from eight healthy volunteers varied substantially both intra- and inter-individually (Figure 2B). Four of the eight healthy volunteers had a greater than 3-fold range in PAR levels over the 3-week sampling time (HV2, 135–408 pg/1×10^7^ cells; HV4, 52–177 pg/1×10^7^ cells; HV5, 73–262 pg/1×10^7^ cells; and HV8, 108–442 pg/1×10^7^ cells); inter-day CVs for individual healthy volunteers ranged from 25% to 68%. PAR levels were also measured in PBMC samples from 14 patients on the NCI Phase 0 trial of ABT-888 [1]. Samples were taken on days −7, −6, −5, and just prior to drug administration (day 1) and showed substantial intra-patient, inter-day variability in PAR levels, with CVs ranging from 1.0% to 26.1% (Table 4). The mean inter-patient CV for the Phase 0 samples was 16.1%. As previously reported, day 1 PAR levels were used as the baseline in the Phase 0 trial [1], [11].

**Table 4:** PAR levels in PBMCs collected from patients during the Phase 0 clinical trial of ABT-888.

	PAR levels (log [pg/1×10^7^ cells])						
Patient	Day -7^a^	Day -6^a^	Day -5^a^	Day 1^a^	Mean	SD	CV (%)
1	2.3	2.5	2.2	2.1	2.3	0.2	7.0
2	2.5	2.4	2.3	2.3	2.4	0.1	4.7
3	2.6	2.7	2.6	2.8	2.7	0.1	3.6
4	3.3	2.9	2.9	3.2	3.1	0.2	6.1
5	NV	1.8	1.8	LLQ	1.8	0.0	1.6
6	2.1	2.1	2.0	2.0	2.1	0.1	3.1
7	2.1	2.7	2.4	2.2	2.3	0.3	11.2
8	2.2	2.3	1.8	1.9	2.0	0.2	11.3
10	3.0	2.4	1.7	2.0	2.3	0.6	26.1
11	2.2	2.1	1.9	2.3	2.1	0.1	7.1
12	2.3	2.2	2.2	2.2	2.2	0.1	3.2
13	1.7	NV	1.7	1.8	1.7	0.0	1.0
14	2.0	2.1	1.7	2.0	1.9	0.1	7.4
					**Mean inter-patient CV (%)**		16.1

Abbreviations: SD, standard deviation; CV, coefficient of variation; NV, no value; LLQ, PAR level below lower limit of quantitation.

a PBMCs were isolated from whole blood collected 7, 6, and 5 days prior to drug administration and immediately before drug administration (day 1).

### Dose-dependent decreases in PAR levels after ex vivo treatment of PBMCs with ABT-888

**Figure 3:**
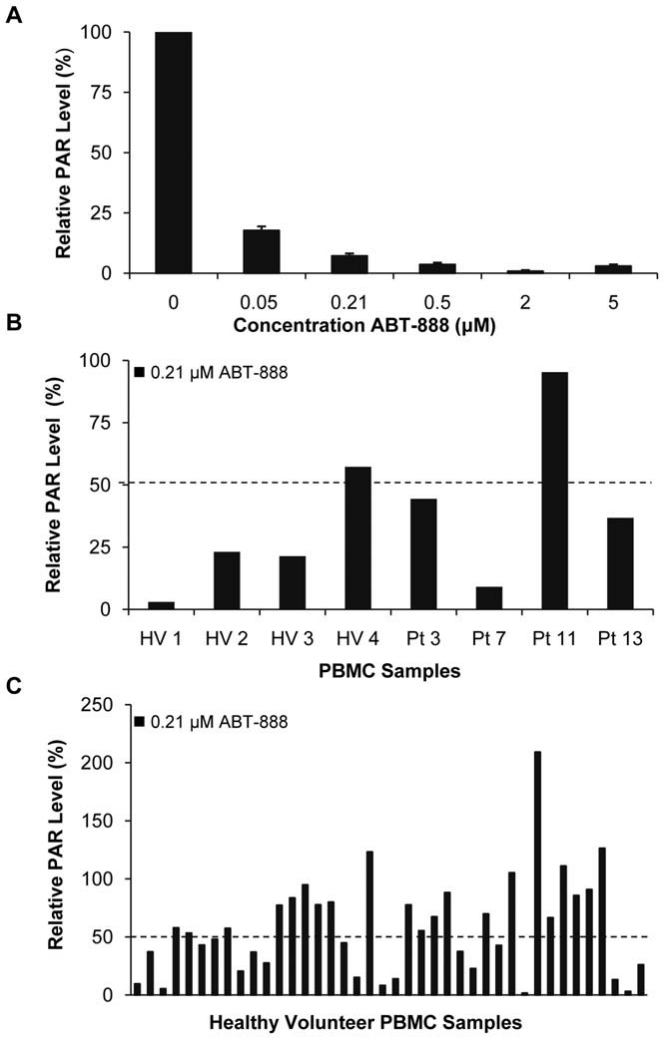
PBMC PAR levels in healthy volunteers and patients with cancer after ex vivo ABT-888 treatment. (A) Pooled PBMCs from healthy volunteers were treated ex vivo for 2 h with increasing concentrations of ABT-888. PAR levels were then determined by PAR immunoassay and normalized (100%) to the vehicle-treated control. Error bars represent standard deviations from three separate experiments. PAR levels were compared between (B) PBMCs from four healthy volunteers (HV) and four patients (Pt) with cancer and (C) 40 individual healthy volunteers. PBMC samples were treated ex vivo with 0.21 µM ABT-888 (the target clinical exposure) for 2 h and PAR levels are reported relative to vehicle-treated controls (100%). Dashed line, 50% reduction.

In preliminary experiments, treating THP-1 human acute monocytic leukemia cells with 0.21 µM ABT-888, the target exposure in the Phase 0 clinical trial [8], resulted in a greater than 90% decrease in PAR levels 2 h after treatment; this inhibition was maintained up to 6 h after exposure (data not shown). To determine the effects of ABT-888 on PBMCs, PBMCs were collected from healthy volunteers, pooled, and treated ex vivo for 2 h with a range of ABT-888 concentrations. Prior to ex vivo treatment, PAR levels were determined for both the individual samples and the pooled PBMC sample; the arithmetic mean of the individual samples matched the pooled sample (data not shown). Ex vivo treatment of PBMCs with ABT-888 resulted in concentration-dependent decreases in PAR levels; treatment with the target clinical exposure of 0.21 µM ABT-888 lowered PAR levels in PBMCs by greater than 90% compared to vehicle-treated controls (Figure 3A).

Ex vivo treatment of individual PBMC samples from four healthy volunteers and four patients with cancer with 0.21 µM ABT-888 resulted in a greater than 50% decrease in PAR levels in three of the four samples from each group (Figure 3B); PAR levels in one sample from a patient with cancer (Pt 11) were not affected by exposure to 0.21 µM ABT-888. Ex vivo treatment of PBMC samples from 40 individual healthy volunteers with 0.21 µM ABT-888 resulted in greater than 50% PAR reduction in 19 (47.5%) of the samples compared to vehicle-treated controls; several donor samples were insensitive to 0.21 µM ABT-888 (Figure 3C).

## Discussion

Use of a validated pharmacodynamic assay to confirm target modulation by molecularly targeted agents can inform drug development decisions early in the clinical evaluation process and has the potential to inform clinical decisions [13], [14]. To this end, we adapted our method for determining PAR levels in tumor biopsies and validated it for use with PBMCs. The Division of Cancer Treatment and Diagnosis offers training and certification on the standard operating procedures for this assay to ensure pharmacodynamic data collected at clinical centers participating in NCI-sponsored clinical trials of PARP inhibitors are accurate and comparable between clinical sites and trials [10]. Using PBMCs as a surrogate for pharmacodynamic effects of PARP inhibitors on tumor has obvious advantages: PBMCs are readily accessible, their collection confers minimal risk to patients, and they allow longitudinal assessment of drug activity over the course of treatment. With our validated PAR immunoassay for PBMCs, we were able to detect PAR in all of the PBMC samples tested; greater than 90% of the samples from healthy volunteers and patients with cancer had PAR levels higher than the lower limit of quantitation. The sensitivity and quantitative range of the PAR immunoassay is feasible for measuring changes in PAR levels in PBMC samples collected during clinical trials. The data obtained may help determine optimal dosing schedules, duration of treatment, and the administration sequence of PARP inhibitors in combination with other agents.

Our initial efforts to model PARP inhibition in mouse models by mirroring clinical procedures have been described previously [8]. One advantage of using human PBMCs for modeling was that they could be treated with PARP inhibitors ex vivo using clinically relevant doses and potentially could serve as an indicator for patient sensitivity to drug. The 0.21 µM concentration of ABT-888 was selected in early studies because it is the plasma concentration associated with a significant reduction in PAR levels in single-dose studies in mouse models and was the target exposure in the Phase 0 clinical trial [8]. If the data from our current and planned Phase I and II clinical trials of PARP inhibitors confirm that PBMCs can serve as a pharmacodynamic surrogate for drug effect on tumor, we may consider pre-enrollment screening in Phase III clinical trials for patients likely to benefit from ABT-888 treatment.

It should be noted that no correlation in PAR levels has been reported between patient tumor and PBMC samples. Although levels of PARP1 expression and/or activity are generally reported to be higher in tumor cell lines than in normal cells [15], [16] and in several primary tumor types, including triple-negative breast cancer, than in syngeneic nonmalignant tissue [17], comparisons of PARP activity or PAR levels in PBMCs to that in tumor tissue are not abundant. One recent publication found no significant difference in either PARP1 expression levels or PARP1 activity in PBMC samples from healthy volunteers and patients with cancer [18]. Our results support these conclusions since we found no significant difference in mean PAR levels in PBMCs from healthy volunteers and patients with cancer. The question of whether the reduction in PAR levels in PBMCs after exposure to ABT-888 predicts reduction in PAR levels in tumor, and whether this reduction is proportional, remains to be addressed. Data from ongoing Phase I and II trials at the NCI will be analyzed in an attempt to answer this question. Subsequent Phase III efficacy trials of ABT-888 will, if warranted, attempt to establish whether absolute reduction or percent reduction in PAR is of greater clinical significance.

Our data indicate that PBMCs from some healthy volunteers are not sensitive to ABT-888. The reasons for this are not known, though we had previously observed a similar phenomenon with a patient in the Phase 0 trial of ABT-888 [1]. In that trial, greater than 50% reduction in PAR was quantifiable in PBMC samples from 11 of 13 patients. One patient experienced no significant reduction in PAR levels in either PBMCs or tumor biopsy after administration of ABT-888, and a PBMC sample obtained from this patient was similarly insensitive to drug treatment ex vivo. The patient's plasma levels of ABT-888 were comparable to the other patients in the dose cohort, and no unique single nucleotide polymorphisms (SNPs) or significant differences in the ratio of PARP1 and PARP2 to poly (ADP-ribose) glycohydrolase (PARG) mRNA expression levels were found that might account for insensitivity to the drug [1]. Lack of correlation between PARP activity, protein level, and polymorphisms has been reported by others [16]. Future ex vivo studies will compare the sensitivity of PBMCs from the same donor to different PARP inhibitors to assess differences in mechanism of action and potency.

To our knowledge, this is the first report of inter-day variability in PAR levels in samples from healthy volunteers. The range in baseline PAR levels measured between all healthy volunteer samples was 39-fold and in patients with cancer was 32-fold, demonstrating a broad heterogeneity inherent in the population. Inter-individual variation in poly(ADP-ribosyl)ation capacity in healthy volunteer PBMCs has been reported previously [19]. While we do not know the reason for the baseline fluctuation in PAR levels measured in healthy volunteers and patients, we are currently conducting flow cytometry and fluorescence microscopy analyses to isolate and identify sensitive subpopulations of PBMCs. In view of the role of PARP in DNA repair in healthy cells and DNA repair–deficient tumors [20], [21], one objective of our Phase II clinical studies of ABT-888 in combination with chemotherapeutic agents is to assess whether prolonged suppression of PARP is biologically necessary or clinically beneficial; a mechanism for measuring PAR levels throughout the course of treatment will be essential for these studies.

PARP enzymes catalyze the poly(ADP-ribosyl)ation of many proteins involved in DNA transcription and repair, chromatin remodeling, and cell death [2]. PARP activation is a characteristic of several pathological conditions and diseases in addition to cancer, and as such, there is considerable interest in evaluating PARP inhibitors for the treatment of diabetic retinopathy, cardiovascular disease, inflammation, and stroke [6], [15]. Using PBMCs as a surrogate for the evaluation of pharmacodynamic effects after treatment allows for a minimally invasive method for determining changes in PAR levels and a means to evaluate longitudinal effects of drug administration. Thus, our validated method for quantifying PAR levels in PBMCs may have broad application in the preclinical and clinical pharmacodynamic evaluation of PARP inhibitors.

## Materials and Methods

### PBMC collection and preparation

Blood samples from healthy volunteers and patients with cancer (various types of solid tumors) at the National Institutes of Health and NCI-Frederick Blood Banks were collected in 8-mL Cell Prep Tubes (Becton Dickinson, Rockville, MD); PBMCs were isolated to determine PAR levels. In addition, four healthy volunteers and four patients with cancer provided serial PBMC samples collected once a week for 3 consecutive weeks. Samples were also collected from 14 patients participating in the Phase 0 trial of ABT-888 (ClinicalTrials.gov identifier: NCT00387608) on days −7, −6, −5, and 1, where day 1 was the first day of drug administration [1], [11]. All patients and healthy donors gave written informed consent for study inclusion and were enrolled on NCI institutional review board–approved protocols. The study was performed in accordance with the precepts established by the Helsinki Declaration. The study design and conduct complied with all applicable regulations, guidance, and local policies and was approved by the NCI institutional review board.

Whole blood samples were gently inverted eight times prior to centrifugation at 1500 x *g* for 30 min at 18°C to 25°C on the “no brake” setting. PBMCs were collected by decanting the buffy coat and interfacing cells into 15-mL conical centrifuge tubes containing PlasmaLyte A, pH 7.4, USP (Baxter Healthcare, Deerfield, IL). Viable cells were counted using a hemocytometer with trypan blue. Cells for the PAR immunoassay were resuspended at a density of 3×10^6^ viable cells/mL in PlasmaLyte A, aliquoted into 1.5-mL screw-capped centrifuge tubes, and then centrifuged again to pellet the cells. The supernatant was aspirated, and the PBMC pellet in the tube was flash-frozen and stored at −80^o^C until use.

### Cell lysate preparation

Frozen cell pellets were suspended in 100 µL of Cell Extraction Buffer (Invitrogen, Carlsbad, CA) per 1×10^6^ cells (1×10^7^ cells/mL), supplemented with protease inhibitor cocktail tablets (Roche Applied Science, Indianapolis, IN) and 1 mM phenylmethanesulfonyl fluoride (Sigma-Aldrich, St. Louis, MO). Lysates were incubated on ice for 30 min prior to adding sodium dodecyl sulfate (Ambion, Austin, TX) to a final concentration of 1%. Tubes were then boiled for 5 min to inhibit intrinsic enzyme activity and stabilize PAR. Cell extracts were snap-cooled in an ice bath and then centrifuged at 10,000 x *g* for 5 min at 4°C. Clarified lysates were assayed immediately, using 25 µL of extract per well in the PAR immunoassay. When specified, extracts were assayed for total protein concentration using a Bicinchoninic Acid (BCA) Protein Assay Kit (Pierce, Rockford, IL) adapted for use in a 96-well plate format according to the manufacturer's instructions.

### Immunoassay for PAR substrates

The validated chemiluminescent immunoassay for PAR using commercially available anti-PAR mouse monoclonal antibody (clone 10H, Trevigen, Gaithersburg, MD) is described in detail elsewhere [8], [10]. Briefly, 100 µL of antibody at a concentration of 4 µg/mL in 0.1 M carbonate-bicarbonate buffer (pH 9.6) was added to each well of a 96-well white microtiter plate and incubated at 37°C for 2 h. Wells were blocked with 250 µL SuperBlock (Thermo Scientific, Waltham, MA) at 37°C for 1 h. Pure PAR polymers (BioMol International, Plymouth Meeting, PA) were serially diluted in SuperBlock to a range of 7.8 to 1000 pg PAR/mL and served as standard controls. PAR standards or cell extracts were loaded in 25 µL volumes plus 50 µL SuperBlock per well, in triplicate, onto each plate and incubated at 4°C for 16±1 h. Next, 100 µL/well of anti-PAR rabbit polyclonal antibody (0.5 µg/mL; Trevigen) diluted with 2% bovine serum albumin (Sigma-Aldrich) in 1X phosphate buffered saline (Invitrogen) supplemented with 1 µL/mL normal mouse serum (Sigma-Aldrich) was added and incubated at 24°C for 2 h. Then 100 µL/well of goat anti-rabbit horseradish peroxidase conjugate (KPL, Gaithersburg, MD) at a final concentration of 1 µg/mL (1∶1000) diluted with 2% bovine serum albumin in phosphate buffered saline supplemented with 1 µL/mL normal mouse serum was added and incubated at 24°C for 1 h. Finally, 100 µL/well of fresh SuperSignal ELISA Pico Chemiluminescent Substrate (Thermo Scientific) was added and the plate immediately read on a Tecan Infinite M200 plate reader (Tecan Systems, San Jose, CA). Relative light unit values were plotted using a PAR analysis template to generate standard curves. Average PAR level, standard deviation, and CV for each PBMC extract were determined from the PAR standard curve. Final PAR readout for each sample was reported as pg PAR/mL of cell extract using the PAR standard curve. Back calculation using PBMC extract dilution (1∶3) resulted in PAR levels reported as pg/1×10^7^ cells.

### Assay specificity, accuracy, and precision validation

As with the PAR immunoassay in tumor extracts, some cross-reactivity was seen by Western blot with the rabbit polyclonal PAR antibody (data not shown) [8]. Bovine serum albumin was again used in the probe and conjugate diluents to absorb this cross-reactivity. For recovery experiments, PAR polymer prepared in SuperBlock was spiked into PBMC extracts with known PAR levels. Expected versus observed PAR recovery was assayed for three paired replicates by two different operators to assess assay accuracy. Assay controls and standards were run on each plate. Pooled PBMC extracts spiked with known amounts of PAR polymer (31.25, 62.5, and 125 pg PAR/mL) plus the assay zero were assayed as unknowns by two operators on two different instruments (Infinite 200 Microplate Reader [Tecan]; Berthold Luminometer [Berthold Detection Systems, Huntsville, AL]) for 3 days. Extracts made from Colo829 human melanoma cell (ATCC, Manassas, VA) extracts were qualified using the PAR immunoassay and used as known dilutions for assay controls. CVs of apparent specimen concentrations based on reading the standard curve are reported except for the assay zero, which is reported as the CV of the instrument. Data were collected during certified assay operator training on the validated PAR immunoassay [10] held by the Division of Cancer Treatment and Diagnosis at NCI-Frederick for longitudinal assessment of assay performance. To allow for longitudinal comparison of PAR assay performance, the average PAR readout for each training date PBMC sample was set at 100% and used to determine relative PAR measured by individual operators.

### PAR recovery

Dilution linearity was tested by diluting PBMC extract into SuperBlock and back-calculating the PAR concentration in the starting material at each dilution tested. PAR polymer was prepared in SuperBlock as for a standard curve determination and was then spiked into a pool of extract made from four PBMC aliquots from four healthy volunteers; the spiked pooled extract was then serially diluted to final concentration of 1000, 500, 250, 125, 62.5, 31.25, 15.625 and 7.8 spiked-PAR pg/mL and assayed at 4^o^C using identical assay reagents. Extracts were pre-diluted in Superblock to 2, 4, 8, and 10 µg total protein/37.5 µL. Extracts were added to wells containing either 37.5 µL of the assay diluent or 37.5 µL of PAR polymer standards in duplicate wells, and then assayed as described previously in the methods section. Assay controls and standards were run on each plate. Each recovery experiment was performed twice, and linear fit was applied to the resulting dilution curve.

### Ex vivo PBMC culture

Aliquots of 1×10^7^ PBMCs collected as described above were cultured in RPMI 1640 media (Invitrogen) in an uncapped 2-mL tube with up to 5 µM ABT-888 (NSC 737664; Abbott Pharmaceuticals, Abbott Park, IL) for 2 h in a humidified 37°C incubator supplemented with 5% CO_2_. ABT-888 was solubilized in sorbitol (105 mg/mL, Sigma-Aldrich) and citric acid (monohydrate; 5.17 mg/mL, Sigma-Aldrich) in sterile water.

### Statistical analysis

Values for mean, median, standard deviation, correlation coefficients, and CV were determined using Microsoft Excel software. PAR levels in PBMCs collected from patients during the Phase 0 clinical trial (Table 4) were log-transformed due to a non-normal distribution. Inter- and intra-assay CVs were calculated to determine assay accuracy. Student's *t*-tests were unpaired, two-tailed tests with the significance level (α) set at 0.05 (95% confidence level). Assay imprecision was calculated as the square root of the sum of the intra- and inter-operator CVs for control sample data collected during the Division of Cancer Treatment and Diagnosis training courses. Control samples from student trainees (19 students over seven training courses) and the course trainer (one trainer in four of the seven courses) were only included in the calculations if they passed quality control criteria established in the standard operating procedure [10]. The Grubb's test with significance level (α) set at 0.05 was used to detect outliers in the trainee CV value results using GraphPad software (GraphPad Software Inc., La Jolla, CA).
